# Supplementary material for: “I Shouldn’t Have to Drive to the Suburbs”: Grocery Store Access, Transportation, and Food Security in Detroit During the COVID-19 Pandemic
Source: Nutrients. 2025 Jul 26;17(15):2441. doi: 10.3390/nu17152441 (PMC12348675; doi:10.3390/nu17152441)
Supplement: Supplementary file 1 [file nutrients-17-02441-s001.zip › nutrients-3727138-supplementary.pdf]

Supplemental Table S1. Sample demographics compared to city of Detroit

Demographics	Sample n (%)	Detroit, MI (Estimated adult population=505,520; Estimated households= 270,446) N (%)
Age		
18-24 years old	16 (2.52%)	67948 (13.44%)
25-34 years old	73 (11.51%)	104175 (20.61%)
35-44 years old	130 (20.50%)	79058 (15.64%)
45-54 years old	139 (21.92%)	79439 (15.71%)
55-64 years old	139 (21.92%)	81184 (16.06%)
65-74 years old	116 (18.30%)	54930 (10.87%)
75 and older	21 (3.31%)	38786 (7.67%)
Gender Identity		
Male	129 (19.88%)	234,953 (46.48%)
Female	510 (78.58%)	270,567 (53.52%)
Transgender, Non-Binary or self-described gender identity	10 (1.54%)	N/A
Senior (≥ 65+) in household	253 (38.57%)	74,053 (27.38%)
Child (< 18) in household	227 (34.60%)	76,807 (28.40%)
Race & Ethnicity		
Non-Hispanic Black	389 (60.59%)	514,845 (76.57%)
Non-Hispanic White	148 (23.05%)	74,015 (11.01%)
Hispanic	43 (6.70%)	52,077 (7.75%)
Non-Hispanic Other and Multiple Races	62 (9.66%)	31,414 (4.67%)
Annual Household Income (Gross)		
< $20,000	254 (39.63%)	89,841 (33.21%)
$20,000-$34,999	155 (24.18%)	52,933 (19.57%)
$35,000-$49,999	93 (14.51%)	40,095 (14.83%)
$50,000-$74,999	62 (9.67%)	39,515 (14.61%)
$75,000 or more	77 (12.01%)	48,062 (17.77%)
Education		
Some high school	55 (8.51%)	92,626 (18.32%)
High school graduate/ GED or Equivalent	119 (18.42%)	166,621 (32.96%)
Some college or Associates/Technical Degree	256 (39.63%)	170,054 (33.64%)
Bachelor's or Postgraduate/Professional Degree	216 (33.44%)	76,219 (15.08%)

Supplemental Table S2. Variance Inflation Factors for Associations Between Grocery Store Type and Transportation

Variable	Model 1	Model 2	Model 3	Model 4
Transportation	1.00	1.04	1.15	1.23
Age		1.01	1.03	1.04
Gender				
Male		1.07	1.12	1.12
Female		1.07	1.08	1.08
Race				
Non-Hispanic White			1.20	1.20
Hispanic			1.08	1.09
Other			1.09	1.09
Income-Poverty-Ratio			1.24	1.46
Snap Participation				1.42
Mean VIF		1.04	1.12	1.19

Supplemental Table S3. Variance Inflation Factors for Associations Between Grocery Store Type and Grocery Store Location

Variable	Model 1	Model 2	Model 3	Model 4
Location	1.00	1.02	1.02	1.03
Age		1.02	1.05	1.05
Gender				
Male		1.07	1.10	1.11
Female		1.07	1.07	1.08
Race				
Non-Hispanic White			1.19	1.19
Hispanic			1.07	1.09
Other			1.09	1.09
Income-Poverty-Ratio			1.11	1.40
Snap Participation				1.33
Mean VIF	1.00	1.04	1.09	1.15

Supplemental Table S4. Variance Inflation Factors for Associations Between Grocery Store Type and Food Insecurity Status

Variable	Model 1	Model 2	Model 3	Model 4
Food Insecurity Status				
Low Food Security	1.13	1.20	1.33	1.34
Very Low Food Security	1.13	1.17	1.44	1.44
Age		1.06	1.06	1.07
Gender				
Male		1.07	1.11	1.11
Female		1.07	1.08	1.08
Race				
Non-Hispanic White			1.22	1.22
Hispanic			1.10	1.10
Other			1.06	1.07
Income-Poverty-Ratio			1.36	1.64
Snap Participation				1.34
Mean VIF	1.13	1.04	1.20	1.24

Supplemental Table S5. Variance Inflation Factors for Associations Between Fruit and Vegetable Intake and Grocery Store Type

Variable	Model 1	Model 2	Model 3	Model 4
Shopping Behavior	1.00	1.04	1.11	1.11
Age		1.01	1.04	1.04
Gender				
Male		1.07	1.10	1.10
Female		1.07	1.08	1.08
Race				
Non-Hispanic White			1.20	1.20
Hispanic			1.08	1.09
Other			1.09	1.09
Income-Poverty-Ratio			1.17	1.44
Snap Participation				1.32
Mean VIF		1.04	1.11	1.16
